# Effectiveness and cost-effectiveness of guided Internet- and mobile-based CBT for adolescents and young adults with chronic somatic conditions and comorbid depression and anxiety symptoms (youthCOACH_CD_): study protocol for a multicentre randomized controlled trial 

**DOI:** 10.1186/s13063-019-4041-9

**Published:** 2020-03-12

**Authors:** Frederike Lunkenheimer, Matthias Domhardt, Agnes Geirhos, Reinhold Kilian, Annabel S. Mueller-Stierlin, Reinhard W. Holl, Thomas Meissner, Kirsten Minden, Morten Moshagen, Ramona Ranz, Cedric Sachser, Doris Staab, Petra Warschburger, Harald Baumeister, COACH consortium

**Affiliations:** 1 Department of Clinical Psychology and Psychotherapy, Faculty of Engineering, Computer Science and Psychology, Institute of Psychology and Education, Ulm University, Albert-Einstein-Allee 47, 89081 Ulm, Germany; 2 Department of Psychiatry and Psychotherapy II, BKH Günzburg, Ulm University, Günzburg, Germany; 3 Institute of Epidemiology and Medical Biometry, ZIBMT, Ulm University, Ulm, Germany; 4 Department of General Pediatrics, Neonatology and Pediatric Cardiology, University of Düsseldorf, Düsseldorf, Germany; 5 Charité University Medicine Berlin, Berlin, Germany; 6 German Rheumatism Research Centre, Berlin, Germany; 7 Department of Psychological Research Methods, Institute of Psychology and Education, Ulm University, Ulm, Germany; 8 Clinic of Child and Adolescent Psychiatry/Psychotherapy, University Hospital Ulm, Ulm, Germany; 9 Department Psychology, Counselling Psychology, University of Potsdam, Potsdam, Germany

**Keywords:** Depression, Anxiety, AYA, Internet- and mobile-based interventions, Chronic somatic conditions, RCT

## Abstract

**Background:**

Adolescents and young adults (AYA) with chronic somatic conditions have an increased risk of comorbid depression and anxiety symptoms. Internet- and mobile-based cognitive behavioural therapy (iCBT) might be one possibility to extend the access to evidence-based treatments. Studies suggest that guided iCBT can reduce anxiety and depression symptoms in AYA. However, little is known about the effectiveness of iCBT for AYA with chronic somatic conditions and comorbid symptoms of anxiety and/or depression in routine care. Evidence on the (cost-)effectiveness of iCBT is essential for its implementation in health care.

**Objectives and methods:**

This multicentre two-armed randomized controlled trial (RCT) aims to evaluate the (cost-) effectiveness of guided iCBT (youthCOACH_CD_) in addition to treatment as usual (TAU) compared to enhanced treatment as usual (TAU+) in AYA aged 12–21 years with one of three chronic somatic conditions (type 1 diabetes, cystic fibrosis, or juvenile idiopathic arthritis). AYA with one of the chronic somatic conditions and elevated symptoms of anxiety or depression (Patient Health Questionnaire [PHQ-9] and/or Generalized Anxiety Disorder [GAD-7] Screener score ≥ 7) will be eligible for inclusion. We will recruit 212 patients (2 × *n* = 106) in routine care through three German patient registries. Assessments will take place at baseline and at 6 weeks, 3 months, 6 months, and 12 months post-randomization. The primary outcome will be combined depression and anxiety symptom severity as measured with the PHQ Anxiety and Depression Scale. Secondary outcomes will include health-related quality of life, coping strategies, self-efficacy, stress-related personal growth, social support, behavioural activation, adjustment and trauma-related symptoms, automatic thoughts, intervention satisfaction, working alliance, and Internet usage. The cost-effectiveness will be determined, and potential moderators and mediators of intervention effects will be explored.

**Discussion:**

iCBT might implicate novel ways to increase the access to evidence-based interventions in this specific population. The distinct focus on effectiveness and cost-effectiveness of youthCOACH_CD_ in patients with chronic somatic conditions, as well as intervention safety, will most likely provide important new insights in the field of paediatric e-mental health. A particular strength of the present study is its implementation directly into routine collaborative health care. As such, this study will provide important insights for health care policy and stakeholders and indicate how iCBT can be integrated into existing health care systems.

**Trial registration:**

German Clinical Trials Register (DRKS), DRKS00017161. Registered on 17 September 2019.

## Background

On average, 15% of children and adolescents suffer from chronic somatic conditions, and the trend is rising [1]. Disabling conditions are—amongst others—diabetes, with 310/100,000 children and adolescents [2], cystic fibrosis, with 8/100,000 [3], and juvenile idiopathic arthritis, which affects about 100/100,000 [4] individuals at the transition to adulthood. Current studies estimate the average annual social/economic costs of cystic fibrosis to be €53,256 (SD 46,589) [5], juvenile idiopathic arthritis €27,634 (SD 28,008) [6], and type 1 diabetes with €3745 (inter-quartile range 1943–4881) [7] in adolescents and young adults (AYA) per patient.

Mental disturbances and disorders are common in this population, particularly depression- and anxiety- related symptoms [8, 9], which in turn are associated with reduced quality of life, reduced treatment adherence, poorer long-term prognosis [10], and increased health service use (outpatient and inpatient, medical/medical claims, and pharmacy claims) [11].

AYA with chronic somatic conditions are confronted with two challenges simultaneously: disease-specific requirements (e.g. medication, visits to a physician, physical limitations, etc.) and age-specific developmental tasks (e.g. development of identity and autonomy) [9]. Hence, a significant part of AYA with chronic somatic conditions are in need of mental health support [12].

Cognitive behavioural therapy (CBT) has been shown to be efficacious in the treatment of depressive disorders [13, 14] and anxiety disorders [15, 16], and can be regarded as first-line treatment in children and adolescents [17]. With regard to symptoms of depression and anxiety in chronic somatic conditions, CBT has also been found to reduce symptoms of depressive disorders in adults with chronic somatic conditions [18–20], while the evidence on the effectiveness of CBT in AYA with somatic-mental health comorbidities is limited. Moreover, the access to treatments is limited. In a German sample, less than 30% of AYA with relevant mental health disturbances used mental health care offers (consulted a psychologist, psychiatrist, or psychotherapist) in the past 12 months [21]. Especially amongst AYA with chronic somatic conditions, the high prevalence rates of mental health problems combined with the very low treatment utilization rate show that methods to identify and disseminate empirically validated treatments of these disorders in health care are necessary [22, 23].

Internet- and mobile-based cognitive behavioural therapy (iCBT) might be a promising approach to overcome this mental health care gap and allow for a timely treatment of comorbid mental burden. Advantages of Internet- and mobile-based interventions include temporal and local flexibility, widespread accessibility, presumed cost-effectiveness, and low-threshold access to address psychological aspects of chronic conditions [24, 25]. iCBT, designed as self-help interventions with no or only very limited therapeutic guidance, can improve depressive symptoms and symptoms of various anxiety disorders in adults [26–29]. Several recent systematic reviews and meta-analyses indicate that iCBT is particularly efficacious when provided as therapeutically guided iCBT [30, 31]. As such, Internet- and mobile-based, therapeutically guided CBT can be as efficacious as face-to-face CBT for the treatment of mental disorders [32]. Thereby, iCBT might not only be efficacious but also safe and cost-effective. A recent meta-analysis of individual participant data showed that guided iCBT for adults with depression is associated with a mean reduced risk for symptom deterioration compared to other control conditions [33]. The results of a systematic review on cost-effectiveness indicate that guided Internet- and mobile-based interventions for the treatment of depression symptoms have the potential to be cost-effective [24]. This has also been shown for anxiety symptoms [34]. Also in this complex subpopulation of patients with somatic conditions, a systematic review reported that computerized CBT could also improve symptoms of anxiety and depression [35]. Another meta-analysis reported small to large effect sizes of iCBT on depression (*g* = −.20), anxiety symptoms (*g* = −.21), and physical health symptoms (*g* = − 1.14) within adults with somatic conditions [36]. Internet- and mobile-based interventions can also be used for the (co-)treatment of chronic somatic conditions, e.g. to increase disease-specific self-efficacy in patients with diabetes (*d* = .23) or improve pain-related disability in patients with chronic pain [37, 38].

When it comes to iCBT for AYA, the evidence is far less comprehensive. Still, current evidence suggests that iCBT can be efficacious in the treatment of anxiety and depression in AYA, again probably with higher effect sizes for guided iCBT compared to completely unguided self-help iCBT [31, 39]. However, whether this general conclusion holds true for the subpopulation of AYA with chronic somatic conditions is largely unknown; there are only a few small efficacy trials with some methodological flaws (e.g. high risk of bias due to inadequate blinding, lack of published protocols, incomplete outcome data) [40]. Thus, the effectiveness and cost-effectiveness of embedding iCBT for depression and anxiety in routine collaborative paediatric somatic care still need to be established.

## Methods/design

### Objectives

This trial aims to evaluate the effectiveness and cost-effectiveness of youthCOACH_CD_, a guided iCBT for AYA with type 1 diabetes, cystic fibrosis, or juvenile idiopathic arthritis.

The primary objective is:

1. To evaluate the short-term effectiveness of iCBT in improving depression and anxiety symptoms, assessed using the Patient Health Questionnaire Anxiety and Depression Scale (PHQ-ADS), at 12 weeks post-randomization (t2) compared to enhanced treatment as usual (TAU+).

Secondary objectives of the study are:

2) To evaluate the middle (t3; 6 months post-randomization) and long-term (t4; 12 months post-randomization) effectiveness of iCBT in improving depression and anxiety symptoms compared to TAU+.
3) To evaluate the short (t2), middle (t3), and long-term (t4) effectiveness of iCBT compared to TAU+ in improving (a) health-related quality of life, (b) coping strategies, (c) self-efficacy, (d) stress-related growth, (e) social support, (f) behavioural activation, (g) adjustment and trauma-focused symptoms, and (h) automatic thoughts and also to evaluate the (i) working alliance and (j) intervention satisfaction in the intervention group.
4) To examine the cost-effectiveness of iCBT compared to TAU+ over a period of 12 months.
5) To evaluate the safety of iCBT by examining potential adverse events compared to TAU+.
6) To explore potential moderators and mediators of intervention effects.

### Study design

**Fig. 1:**
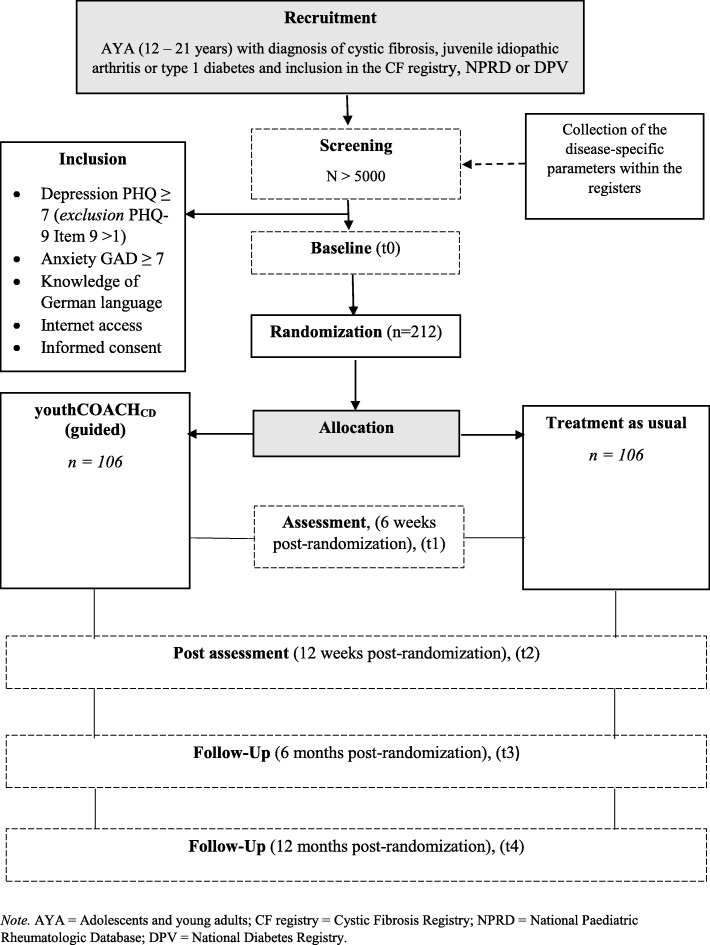
Flow chart of inclusion and study procedure (youthCOACH_CD_ = Internet- and mobile-based cognitive behavioural therapy)

This is a two-armed, multicentre parallel randomized controlled trial (RCT) comparing iCBT to TAU+. The intervention group receives iCBT in addition to treatment as usual (TAU). Assessments will take place starting with mental health screening in paediatric units followed by five assessment points over one year: baseline (t0), 6 weeks (t1), 3 months (t2), 6 months (t3), and 12 months (t4) post-randomization. The flow chart of the study design is shown in Fig. 1.

This clinical trial has been approved by the Ethics Committee of Ulm University (number 292/18) and will be conducted and reported in accordance with Consolidated Standards of Reporting Trials (CONSORT) statements for RCTs [41, 42] as well as the guidelines for executing and reporting Internet intervention research [43]. This trial will be monitored by an independent Data and Safety Monitoring Board (DSMB; CK, LK, CPS) and has been carefully checked for data protection compliance. This study protocol is in accordance with the Standard Protocol Items: Recommendations for Interventional Trials (SPIRIT) guideline [44]. The SPIRIT checklist is provided as Additional file 1.

### Participants and procedure

#### Inclusion and exclusion criteria

AYA between 12 and 21 years of age with type 1 diabetes, cystic fibrosis, or juvenile idiopathic arthritis and elevated anxiety and/or depression symptoms (Generalized Anxiety Disorder Screener, GAD-7 [45] and/or PHQ-9 [46] score ≥ 7) with Internet access and a basic knowledge of the German language are eligible for inclusion. AYA with increased risk of suicidality at screening (PHQ-9 Item 9 > 1) will be excluded for ethical and safety reasons and will receive adequate care within the ongoing clinical routine. Prior to participation, consent to study participation must be given by the AYA or, for adolescents under the age of 16, by a person with custody. As this is a pragmatic RCT, there are no further exclusion criteria.

#### Recruitment

Recruitment start is scheduled for October 2019. The iCBT with its associated independence of space and time enables a recruitment strategy throughout Germany. Anxiety and depression will be screened as part of the clinical routine in hospitals, clinics, medical practices, and medical centres across Germany where AYA with type 1 diabetes, cystic fibrosis, or juvenile idiopathic arthritis receive medical treatment. These clinical institutions are all organized within three well-established German patient registries, the National Paediatric Rheumatologic Database (NPRD) [47], the National Diabetes Registry (DPV) [48], and the Cystic Fibrosis (CF) Registry [49]. The screening data will be gathered in clinical centres and administered within these patient registries. AYA receive feedback on their mental well-being by their health care provider in charge. If AYA show anxiety and/or depression symptoms (GAD-7 [45] and/or PHQ-9 [46] score ≥ 7) without elevated suicidality (PHQ-9 Item 9 ≤ 1), the respective patient is informed by the treating physician at the clinical centre and invited to participate in the study in addition to receiving standard care/TAU. The informed consent will be obtained at the clinic by the attending physician, and all further recruitment activities will be conducted by researchers at Ulm University. For each randomized AYA the respective clinical unit receives €230 as financial compensation for its recruitment effort. Additional study information is provided on https://coach.klips-ulm.de.

#### Randomization

The participants are randomized by the program Sealed Envelope (www.sealedenvelope.com) in an allocation ratio of 1:1 using block randomization. The block size is 6, 8, 10 participants per block and stratified according to the three chronic somatic conditions. Stratification is performed due to the strongly varying prevalence of chronic somatic conditions. Only RR, who is not involved otherwise in the trial process, will conduct the randomization process. AG, FL (study administration), and MM (statistical evaluation) are blinded and do not receive any information about the group allocation of study participants. The blinded members of the study team do not have access to documents showing group membership of participants.

### Study interventions

#### Intervention condition

The intervention group receives the iCBT plus treatment as usual (TAU) and is compared to a control group, which receives TAU+. youthCOACH_CD_, developed by the Department of Clinical Psychology and Psychotherapy (FL, AG, MD, HB) consists of an introductory session with information about the online-based intervention and seven modules of approximately 50 to 70 min processing time each. Modules can be repeated as often as desired. The modules cover the topics motivation and resources, behavioural activation, understanding and coping with anxiety and depression, emotion regulation, communication, and social support as well as relapse prevention. The intervention is presented via information texts, videos, audio recordings, photos, metaphors, and therapeutic homework. The content of the intervention is based on CBT principles for depression and anxiety, including elements of psychoeducation, individual resources of the AYA, active coping (problem solving), restructuring of stressful disease-related thoughts, communication training, relaxation, and behaviour activation to model adaptive coping strategies.

The content of the intervention (see Table 1) is developed for AYA and related to typical challenges and tasks of a life with a chronic somatic condition. The intervention is explained and presented in a youth-friendly manner. To improve patient adherence, interactive elements (e.g. conditional contents, certificates) are implemented, and reminders of the weekly intervention lessons as well as homework assignments are forwarded via a mobile app. The theoretical skills learned in youthCOACH_CD_ can be tested in practice and transferred into everyday life through homework (e.g. diaries), also via a mobile app. At the beginning of the intervention, participants are informed to receive daily reinforcing prompts via a mobile app during the intervention period. The reminders are sent automatically after each module has been completed and coordinated with intervention content to transfer the learned skills into everyday life of the participants. The mobile-based reminders aim to increase motivation of participants, remind participants to complete homework assignments, and repeat intervention content. The software solutions for the iCBT intervention and the mobile app are both provided by Minddistrict (www.minddistrict.com). Access to the iCBT intervention and the app is granted through an individual username-password combination and will be available on a 24/7 basis.

**Table 1:** Intervention content, based on cognitive behavioural therapy (CBT)

	Content	
Introduction: Welcome!	Dealing with online training Schedule, structure, and content of online training Introduction of the e-Coach	
1. Get to know your strengths!	Creating goals Becoming aware of strengths and abilities Use of own traits Techniques to build robust self-esteem Dealing with challenges/learning problem-solving skills	Mood diary
2. Become active!	Psychoeducational information on the relationship between mood and behaviour Exploring activities that give pleasure Daily structuring to reduce stress Integration of physical activity into daily life	
3. Overcoming fears	Psychoeducational information on anxiety Reduction of anxiety	
4. Learn to deal with bad moods	Psychoeducational information on depression Recognition and handling of rumination and dysfunctional thoughts Activation	
5. Recognize and understand very strong feelings!	Psychoeducational information on emotions Acceptance of emotions Dealing with very strong feelings (e.g. skills, relaxation techniques)	
6. Together we are stronger!	Social competence training Communication skills Perception of social support Increasing personal responsibility	
7. You did it!	Summary of the learned contents Coping strategies and support Information on further treatment options regarding mental health	

#### Guidance

The intervention is therapist-guided by e-Coaches, who report semi-standardized feedback after each completed module on the intervention platform to the participant using an e-Coach manual. The e-Coach manual is standardized to guarantee protocol adherence by the e-Coaches. e-Coaches are graduates of a Master’s Degree in Psychology or Pedagogy, who are enrolled in a training program in CBT with children and adolescents, closely supervised by licensed psychotherapists. The feedback content matches the participants’ tasks and is aimed to support adherence to treatment. The feedback also includes positive reinforcement to motivate participants to continue the intervention. For further questions, participants and e-Coaches can communicate via the intervention platform. e-Coaches will also send reminders to participants who do not complete intervention modules at the scheduled time via e-mail and telephone. The weekly schedule for processing a module is set by the participants themselves at the end of the previous module. In order to promote commitment, the intervention is blocked for the participant after three reminders without the participant reacting. For participants who indicate that they wish to continue, youthCOACH_CD_ will be reactivated. Table 2 shows the intervention structure and implementation.

**Table 2:** Intervention structure, technical implementations, and support

Intervention structure	
• Seven weekly modules (50-70 min.)	
Implemented elements	
• Guidance by trained and supervised e-Coaches (graduates of a Master’s Degree, who are enrolled in a training program in CBT with children and adolescents)	
• One feedback message per module by e-Coaches	
• Reminders by the e-Coach if the modules are not processed at a date set in advance by the participant to promote adherence	
• Deactivation and reactivation when modules are not processed and three unsuccessful reminders	
• Answers to additional questions from e-Coaches and the study team	
• Tight security procedure for suicidal clues	
• Weekly challenges/ homework	
• Diaries via a mobile app with daily reminders	
• Information given by text and videos	
• Audio guided exercises	
• Patient examples written and presented as audio recordings	
• Metaphors, quizzes and conditional content	
• Modules can be repeated as often as desired	

#### TAU+

TAU+ includes all routine care services for somatic and mental health problems. TAU+ can vary depending on the clinic, location, care options, and barriers of care as well as the specific needs of AYA. TAU is supplemented (enhanced, +) by screening for symptoms of anxiety and depression and by information letters on evidence-based mental health care options.

TAU+ is not defined a priori, but is recorded alongside the health economic evaluation and can thus be described post hoc descriptively.

### Sample size/power calculation

A clinically significant effect of at least *d* = .50 for the mean difference between the groups is assumed, which refers to the severity of the primary outcome, i.e. a PHQ-ADS sum score [50] at 12 weeks post-randomization (t2). The assumption for the clinically significant effect is based on a meta-analysis by Ebert and colleagues, who reported a moderate to high effect size of *g* = .72 (95% confidence interval [CI] .55–.90) for Internet and computer-based CBT in the reduction of depressive and anxiety symptoms in youth [39], based on trials comparing Internet- and computer-based CBT to non-active control conditions (waitlist; placebo). However, given that TAU usually results in higher effect sizes compared to waitlist control groups, we regard a lower than *g* = .72 effect size as clinically relevant. The sample size calculation was carried out by an external statistician from the Department of Psychological Research Methods at Ulm University (MM) based on a power of .90 and α < .05 (two tailed) considering the cluster structure (intra-class correlation [ICC] = .02) of the different prevalence numbers of these chronic somatic conditions, resulting in 166 participants equally randomized to both conditions (2 × *n* = 83). According to a meta-analysis, studies evaluating the efficacy and effectiveness of computer-based psychological treatments for depression with therapeutic support reported a drop-out rate of approximately 28% [51]. For this reason, the sample is increased by 28%, resulting in a total of *N* = 212 participants (2 × *n* = 106).

Various measures will be taken to minimize study drop-outs. Participants receive €10 as an expense allowance at all psychometric assessment points. After successful completion of all assessments, participants can also take part in a prize draw. Moreover, in order to avoid drop-outs in the intervention group, youthCOACH_CD_ was designed to persuade the target group of AYA using e.g. multimedia features such as videos and audios as well as prompts and reminders [52].

### Assessments and outcomes

#### Data collection methods

The data from screening, outcome measurements, and economic measurements are collected from patients (patient-reported outcome) and from their caregivers/adult reference persons in tablet questionnaires (screening) or in online questionnaires. Medical record data of the participants are collected by physicians and are administered via the patient registers. The data is linked by a patient code (ID patient) and a hash key, in order to combine the data sources in a pseudonymized manner. The research data are stored on a protected cloud using encryption software. Detailed study management protocols and lists are used to monitor data collection. This allows interruptions and protocol deviations to be detected.

For an overview of instruments at screening, baseline (t0), 6 weeks (t1), 3 months (t2), 6 months (t3), and 12 months post-randomization (t4), see Table 3.

**Table 3:**
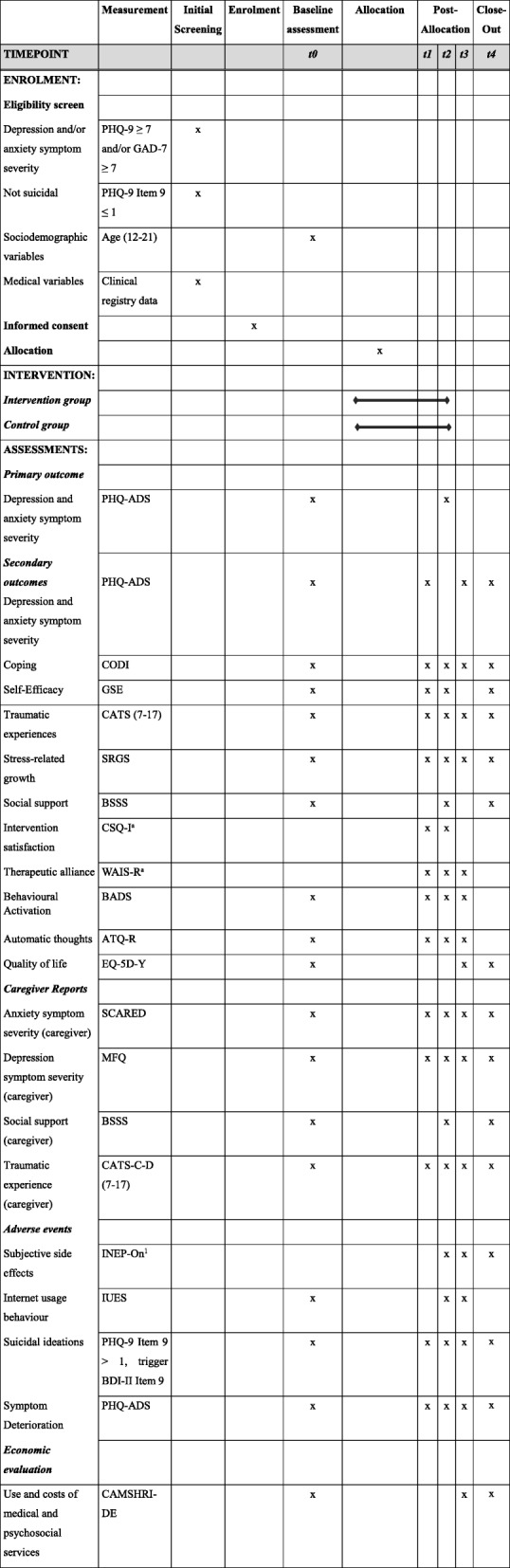
SPIRIT schedule of enrolment, intervention, and assessments

*Notes:* t0 = baseline, t1 = 6 weeks, t2 = 3 months, t3 = 6 months, t4 = 12 months, *PHQ-9* Patient Health Questionnaire, *GAD-7* Generalized Anxiety Disorder Questionnaire, *PHQ-ADS* Patient Health Questionnaire Anxiety and Depression Scale, *CODI* Coping with a Disease, *GSE* General Self-Efficacy Scale, *CATS (7–17)* Child and Adolescent Trauma Screen, *SRGS* Stress-Related Growth Scale, *BSSS* Berlin Social Support Scales, subscale “Actually Received Support”, *CSQ-I* Client Satisfaction Questionnaire adapted to Internet-based interventions, *WAI-SR* Working Alliance Inventory-Short Revised, *BADS* Behavioral Activation for Depression Scale, *ATQ-R* Automatic Thoughts Questionnaire-Revised, *EQ-5D-Y* EuroQol Five-Dimensional Questionnaire-Youth, *SCARED* Screen for Child Anxiety Related Emotional Disorders, *MFQ* Mood and Feelings Questionnaire-Caregiver, *CATS-C-D (7–17)* Child and Adolescent Trauma Screen-Caregiver, *INEP-On* Inventory for recording negative effects of online interventions, *IUES* Internet-Use Expectancies Scale, *BDI-II* Beck Depression Inventory-Revised, *CAMHSRI-DE* Child and Adolescent Mental Health Services Receipt Inventory

^a^Recorded in intervention group only

#### Inclusion criteria and outcome assessments

##### Depression and/or anxiety symptom severity

As part of clinical routine, AYA are screened for symptoms of depression and anxiety. The screening questionnaires are the GAD-7 [45] and the PHQ-9 [46] with a defined cut-off score of ≥ 7 in either of the questionnaires [53].

The German version of the GAD-7 is a self-report anxiety questionnaire. It consists of seven items on a 4-point scale and can be scored from 0 = “not at all” to 3 = “nearly every day”. Good internal consistency (α = .79–.91) [54] and successful usage in adolescents are reported [55].

The German version of the PHQ-9 is administered as a screening inventory to detect depressive symptoms. The PHQ-9 consists of nine items on a 4-point scale with a rating scale ranging from 0 = “not at all” to 3 = “nearly every day”. The computerized version (α = .88) of the PHQ-9 shows an equally high internal consistency as the paper-pencil version (α = .89) [56].

##### Sociodemographic variables

As demographic data, we collect date of birth, gender, relationship status, type of school, grade of school or vocational training, and occupation.

##### Medical variables

The collection of medical data takes place at the time of screening at the respective clinical centre. If the relevant parameters were not collected for screening, the closest examination is used at the time of screening; if two examinations are equally far apart, the previous examination is used, maximum one year away from screening. The following medical data of the participants are collected in the disease-specific registers depending on the chronic somatic condition:

1. *Type 1 diabetes*. HbA1c, number of severe hypoglycaemia (with and without coma) and diabetic ketoacidosis (DKA) events, continuous subcutaneous insulin infusion (CSII)/intensified conventional therapy (ICT), insulin dose per day, self-monitoring of blood glucose (SMBG), continuous glucose monitoring (CGM)/flash glucose monitoring (FGM), diabetes education, and celiac disease
2. *Cystic fibrosis*. Forced expiratory volume in 1 second (FEV 1), diabetes, pancreatic status, liver cirrhosis, pseudomonas infection, number of exacerbations, number of antibiotic therapies, and allergic bronchopulmonary aspergillosis (ABPA)
3. *Juvenile idiopathic arthritis*. Physician’s global assessment of disease activity (numeric rating scale [NRS] 0–10) [57], active joint count, patient/parent global assessment of overall well-being (NRS), the clinical 10-joint Juvenile Arthritis Disease Activity Score [58], functional status (assessed by the Childhood Health Assessment Questionnaire) [59], C-reactive protein (CrP) and juvenile idiopathic arthritis category (systemic arthritis, persistent oligoarthritis, extended oligoarthritis, negative rheumatoid factor polyarthritis, positive rheumatoid factor polyarthritis, enthesitis-related arthritis, psoriatic arthritis, other arthritis).

### Outcome measurements

#### Primary outcome: depressive and anxiety symptom severity

The primary outcome is the combined depressive and anxiety symptom severity at t2, assessed with the PHQ-ADS [50]. Depression and anxiety symptoms at all other assessments will be considered as secondary outcomes. PHQ-ADS is the combined sum score of the questionnaires GAD-7 and PHQ-9 as a composite measure of depression and anxiety, with good internal consistency (α = .88 to .92) [50].

#### Secondary outcomes

##### Coping with chronic health conditions

The Coping with a Disease (CODI) questionnaire for children and adolescents with chronic health conditions [60] is a self-report questionnaire to assess coping strategies of children and adolescents (ages ranging from 8 to 18 years) with chronic health conditions. The CODI questionnaire includes 29 items on a 5-point scale with a rating scale ranging from 1 = “never” to 4 = “all the time”. The questionnaire shows an internal consistency depending on the domain between α = .69 and α = .83 [60].

##### General perceived self-efficacy

General perceived self-efficacy represents a personal coping resource with predictive value for well-being and a constructive coping with life [61, 62]. It serves the personal assessment of one’s own competence, generally to cope with difficulties and barriers in daily life successfully. The reported psychometric parameters of the General Self-Efficacy Scale (GSE) are satisfactory. The internal consistency ranges from α = .80 to .90 for various German samples [63]. The 10 items of the GSE can be rated on a 4-point scale from 1 = “is not true” to 4 = “is absolutely right”.

##### Traumatic experiences and personal growth

The symptom list of the Child and Adolescent Trauma Screen (CATS) for ages 7–17 years is used to observe the changes within treatment in psychological stress caused by traumatic events due to a chronic somatic condition [64]. The questions about trauma-specific symptoms are introduced by the request that the AYA refer to the currently most stressful event with regard to their chronic condition and describe it briefly. The severity of the symptom scale can be rated from 0 = “never” to 3 = “almost always”. Limitations of the functional level in different areas of life are also assessed. In all three countries where the scale has been validated (the USA, Germany, Norway), the 20-item symptom score of the self-report proved good to excellent reliability between α = .88 and α = .94 [64].

The Stress-Related Growth Scale (SRGS) serves to observe the changes that occur during treatment in relation to the individual development caused by the chronic disease. The construction of the SRGS [65] followed the theoretical concept of coping resources of Schaefer and Moos [66]. The present study uses the 15-item short form (α = .89) adapted to the specific population of AYA with a chronic somatic disorder. The items are answered on a 3-point scale with the values 0 = “not at all”, 1 = “something”, and 2 = “a lot” [67].

##### Social support in coping with the disease

The Berlin Social Support Scales (BSSS) [68] differ from other questionnaire methods for social support by their multidimensional approach, that is, by their cognitive and behavioural aspects. AYA are asked whom they relate their answers to. The original questionnaire consists of six scales (Perceived, Received and Achieved Support, Need and Search for Support, Protective Buffering). In this study the subscale “Actually Received Support, Recipient” is used. The subscale includes 11 items plus a general score, and the four-step answer format ranges from “strongly disagree” to “strongly agree”. The subscale together with the general score shows an internal consistency of α = .83 [68].

##### Intervention satisfaction

Participants’ satisfaction with the Internet-based intervention will be assessed with the Client Satisfaction Questionnaire adapted to Internet-based interventions (CSQ-I) [69]. The self-report questionnaire consists of eight items that are rated on a 5-point Likert scale from 1 = “does not apply to me” to 4 = “does totally apply to me”. The scale shows a very good reliability, indicated by McDonald ω values of .93 to .95. The CSQ-I will be used during the intervention (t1 and t2), only for participants receiving youthCOACH_CD_.

##### Therapeutic alliance

In the present study, therapeutic alliance between client and e-Coach/the Internet-based intervention will be assessed with an adapted version of the Working Alliance Inventory-Short Revised (WAI-SR) questionnaire [70]. The 12-item self-report questionnaire is rated on a 5-point Likert scale (1 = “rarely” to 5 = “all the time”). For the German version, internal consistencies between α = .81 and α = .91 were reported for the three subscales (Agreement on Tasks, Agreement on Goals, and Development of an Affective Bond) and an internal consistency between α = .90 and α = .93 for the total score [71]. The WAI-SR will be completed by the intervention group only.

##### Behavioural activation

The Behavioral Activation for Depression Scale (BADS) [72] examines the role of controlling aversive stimuli and avoidance behaviour in depression. As such, it observes changes in activation within treatment. The BADS consists of 25 questions, each rated on a 7-point scale ranging from 0 = “not at all” to 6 = “completely”. The total score demonstrated acceptable internal consistency (α = .79) [73].

##### Automatic thoughts

The Automatic Thoughts Questionnaire-Revised (ATQ-R) [74] records the influence of the intervention on automatic thoughts. The German version of the questionnaire consists of 21 items and can be rated on a 5-point scale between “not at all” and “all the time” [75]. The internal consistencies of the scales of the German version are between α = .75 and α = .89. All scales showed significant correlations with depressiveness and differentiated between adolescents aged between 11 and 16 years with higher and lower depressive symptom severity [75].

##### Quality of life

The generic EuroQol Five-Dimensional Questionnaire (EQ-5D), measuring quality of life, is a short instrument that provides information on health states as a basis for the estimation of quality-adjusted life years (QALYs) [76]. A version for respondents aged 8 to 18 years is the EQ-5D-Youth version (EQ-5D-Y) [77, 78], developed based on the standard EQ-5D [79]. The available utility value sets for adults have been found to be not applicable to children and adolescents [80]. In the absence of a utility value set for children and adolescents in Germany [77], the health states will be valued by means of the visual analogue scale (VAS) of the EQ-5D-Y. The EQ-5D-Y consists of five items and can be evaluated by a 3-point scale (“no problems”, “some problems”, “a lot of problems”). The EQ-5D-Y dimensions were found to be reliable on test-retest (in 69.8–93.8% of Italian youths and in 86.2–99.7% of Spanish respondents) [79]. The EQ-5D-Y not only serves as a secondary outcome, but is also used for economic evaluation.

#### Caregiver reports

##### Depressive and anxiety symptom severity

In order to be able to record the severity of depression and anxiety symptoms not only in self-judgment but also in caregiver reports, a caregiver/adult reference person of the participant is asked to rate the Screen for Child Anxiety Related Emotional Disorders (SCARED) [81] questionnaire and the Mood and Feelings Questionnaire (MFQ) [82].

The SCARED, which examines the third-party rating of anxiety symptoms of the last 3 months, consists of five items and is scored on a 3-point scale from “not true or rare” to “accurate or frequent”. The scale has an acceptable to good internal consistency (α = .74 to α = .93) [83, 84].

The MFQ parent report of depressive symptoms is useful both for preliminary screening and to monitor change in symptomatology [82]. The MFQ short version consists of 13 items, which are rated by a reference person on a 3-point scale from “not true” to “true”. The internal consistency of the original scale is good, with α = .91 to α = .96 [85, 86].

##### Social support

The BSSS [68] will be answered by AYA participants (more information can be found in the section “Secondary outcomes”) and caregivers. The BSSS caregiver report is used to identify how the caregivers assess their own social support for the adolescent. Internal consistency for the 11-item scale “Actually Received Support” is good, with α = .75 [68].

##### Traumatic experiences

Whether the treatment has an influence on the effects of traumatic experiences due to the chronic somatic condition is explored by the stress symptom list of the Child and Adolescent Trauma Screen-Caregiver (CATS-C-D) for AYA aged 7–17 years, from the point of view of the caregivers. The questions about trauma-specific symptoms are introduced by the request that the caregivers refer to the currently most stressful event for the respective AYA with regard to their chronic somatic condition and describe it briefly. The severity of the symptom scale can be rated from 0 = “never” to 3 = “almost always”. The 20-item symptom score of the observer report has proven good reliability with α = .87. Limitations of the functional level in different areas of life are also assessed [64].

#### Adverse events

##### Subjective side effects

Possible unwanted/negative effects of the intervention (t2, t3, and t4, only for participants receiving youthCOACH_CD_) are assessed by the 23 item Inventory for Recording Negative Effects of Online Interventions (INEP-On). This is an adapted version of the Inventory for recording negative effects of psychotherapy that is specifically adjusted for online interventions [87]. Eleven items are rated on a 7-point bipolar scale (− 3 = “better; is totally true” to + 3 = “worse; is totally true”) and 12 items on a 4-point Likert scale (0 = “no agreement at all” to 3 = “total agreement”). The original scale shows a high internal consistency of α = .85 [87].

##### Symptom deterioration

The PHQ-ADS [50] is not only used as a primary or secondary outcome, depending on the assessment time point, but also to determine depression and anxiety symptom deterioration.

##### Internet usage behaviour

In order to explore the change in Internet usage due to treatment, the questionnaire Internet-Use Expectancies Scale (IUES) [88] is used. The IUES consists of eight items with a two-factor structure, that is positive and avoidance expectancies. The items can each be rated on a 6-point Likert scale. Both factors have good reliability (“positive expectancies”, Cronbach’s α = .83 and “avoidance expectancies”, Cronbach’s α = .76) [88]. In addition to the questionnaire, open questions are asked about the duration of use for the Internet and the smartphone, either privately or educationally/professionally.

##### Suicidal ideations

Notwithstanding that suicidality is defined as an exclusion criterion, suicidal ideation might occur during the course of the study and will therefore be monitored closely by means of the PHQ-9 Item 9 at all assessment points (t0–t4). A score ≥ 1 on the suicidality item of PHQ-9 (“Thoughts that you would be better off dead or of hurting yourself in some way?”) leads to the Beck Depression Inventory-Revised (BDI-II) [54] suicidality item (Item 9; BDI-II item = 0: “I’m not thinking of harming myself.”, BDI-II item = 1: “I have thoughts of killing myself, but I would not carry them out”, BDI-II item = 2: “I would like to kill myself”, BDI-II item = 3: “I would kill myself if I had the chance”) [89]. A score ≥ 1 on the BDI-II suicidality item results in a standardized suicide prevention protocol.

Participants screening positive (BDI-II Item 9 ≥ 1) receive an online information letter with detailed information on available and appropriate health services with the advice to use professional help. Participants are informed that youthCOACH_CD_ is not optimized for suicidal ideation management.

In the case of BDI-II Item 9 ≥ 2, participants automatically receive, in addition to the information letter with detailed information on available and appropriate health services, an individual safety plan to fill out (numbers of personal contacts, counselling, medical on-call and emergency numbers), and the project team will follow a detailed, stepped suicide prevention protocol. These participants will be contacted by one of three licensed (child and adolescent) psychotherapists via telephone and interviewed in regard to suicidality. In case of acute suicidality, participants are directed to appropriate on-site mental health care services, when they are compliant. In case of acute suicidality and perceived non-compliance, contact details of participants will be immediately transferred to emergency services in order to secure the safety of participants and initiate appropriate crises intervention; non-compliant participants will be subsequently excluded from the trial. Participants who do not indicate acute suicidal ideations and express compliance will proceed with the study routine.

In order to counteract unexpected side effects or to be able to react when they occur, it is possible for the study participants to contact the study team during working hours by telephone or e-mail. Participants are referred to a nationwide and 24/7 emergency doctor’s telephone hotline and receive detailed information about treatment options. If participants indicate suicidality, the above-mentioned prevention protocol takes effect.

#### Economic evaluation: use and costs of medical and psychosocial services

The Child and Adolescent Mental Health Services Receipt Inventory (CAMHSRI-DE) will be applied to collect information on the clients’ use of health and social services. The extent of service usage is determined on the basis of frequency and duration for eight service categories (inpatient medical services, outpatient medical services, social services—inpatient and outpatient, other inpatient services, school-based services, school types, and medication intake) [90]. The frequency and duration for each health care service will be interpolated for a reference period of 6 months. The CAMHSRI-DE is based on the Client Sociodemographic and Service Receipt Inventory (CSSRI) [91, 92], adjusted for children and adolescents with mental health problems in the German health care system [90]. Costs of service use will be estimated on the basis of unit cost information [93].

### Statistical analyses

All statistical analyses are processed by a biostatistician who is blinded with regard to group assignment. Patterns of missing data will be examined. Multiple imputation with predictive mean matching will be performed to account for missing data. All analyses will be conducted on a two-sided level of significance (α = .05). Participant characteristics will be described descriptively. Rates of patient-reported adverse events will be compared.

### Effectiveness analysis

There will be no interim evaluation of the primary outcome. All statistical analyses will be performed based on the intention-to-treat (ITT) principle. Additional per protocol analyses will be conducted in order to examine the effects of youthCOACH_CD_ in case of patients adhering to the intervention protocol. Participants who completed at least 80% of the intervention are defined as intervention completers (= per protocol).

The primary outcome will be analysed using a hierarchical linear model to account for clustering with the PHQ-ADS score at t2 as dependent variable and the baseline value as covariate, adjusting for sex, age, and chronic somatic condition. Standardized mean differences and 95% CIs will be calculated to measure the between-group effect size at post-treatment (t2) and follow-up (t3, t4). Secondary outcomes will be analysed accordingly.

Exploratory mediation and moderator analyses involving the primary and secondary outcomes as well as demographic data will be conducted. Moderator and subgroup analyses will be attempted in case of a sufficiently large sample size.

### Health economic evaluation

Cost-effectiveness analyses of the iCBT intervention will be estimated from the societal perspective by means of the net benefit method [94–96]. The incremental cost-effectiveness ratio (ICER) will be computed to estimate the maximum willingness to pay (MWTP) *λ* necessary for the gain of one QALY by iCBT in comparison to TAU+. The stochastic uncertainty of the ICER will be estimated by non-parametric bootstrapping [95, 96]. The interpretation of results is based on the cost-effectiveness acceptance curve using a range of MWTP thresholds between 0 and 100,000 € [95–97].

## Discussion

The aim of this study is to examine the (cost-)effectiveness of youthCOACH_CD_, an iCBT for AYA with a chronic somatic condition and comorbid depressive and/or anxiety symptoms.

The innovative aspects of the study are the following: (1) youthCOACH_CD_ is implemented directly into routine medical care. As such, the present study will provide important insights for health care policy on how to integrate iCBT into our health care systems. (2) Studies show that AYA between the ages of 12 and 25 spend an average of 22 h a week online with an increasing tendency to communicate, play, or entertain. Four out of five of 12- to 17-year-olds (80.6%) have used the Internet daily in the last 12 months [98]. Only 4.6% of adolescents at this age do not make use of the Internet. The fact that AYA grow up as digital natives could make online use of an intervention well accepted.

Continuing with advantages, the methodical strengths of the study are as follows: (3) The area-wide recruitment via the German patient registers enables a representative sample and facilitates generalization. (4) With a target sample of 212 adolescents, the study will be high-powered (1 – β = .90). (5) In addition to self-report questionnaires, the study also includes caregiver self-reports on their perception of participants’ conditions. This provides further insights into the effectiveness of iCBT. (6) Not only the effectiveness of youthCOACH_CD_, but also undesirable potential adverse events will be investigated in detail in this study. (7) As one of as yet only a few clinical trials on psychological interventions for AYA with chronic somatic conditions and symptoms of depression and anxiety, the present RCT also comprises cost-effectiveness analyses. (8) Finally, in addition to the psychological parameters, medical data are collected, which are linked to each other and thus allow deeper insights into comorbidity.

The study has several possible obstacles and limitations. First, adolescents aged 12 to 16 years need the informed consent of the custodial persons to participate in the study. This legal constraint might lead to a left-skewed age distribution of the study participants. By adjusting the covariate baseline to age, sex, and chronic somatic disease, this possible limitation is taken into account in the statistical analysis.

Second, intervention and study adherence might be limited in AYA, as has been reported before [99]. Therefore, a drop-out rate of 28% was established. As a counterstrategy we developed our intervention based on persuasive design aspects and provide it therapeutically guided—measures that are associated with improved intervention adherence [51, 100]. Additionally, participants receive a monetary compensation for their study efforts, which will likely increase study adherence.

Third, participant inclusion is based on depression/anxiety symptoms and not on mental disorder status. The reason for this is that the assessments are based on participants’ self-report, which is complemented by caregiver reports. Therefore, participants, caregivers, and physicians of the recruitment supporting clinics are informed that youthCOACH_CD_ is designed as a (guided) self-help intervention that does not replace on-site mental health care according to existing guidelines in case of manifest mental disorders.

Fourth, given the nature of the present trial, participants need to go through several self-report assessments. This might bias effectiveness results in two ways. On the one hand, the assessments might lead to a self-selection of participants with only those being randomized who are determined enough to finalize a comprehensive baseline assessment. At the same time, reminders on the follow-up assessments might have an intervention adherence facilitating effect, reminding participants not only to conduct the assessments but to continue the intervention or reminding them e.g. of learned intervention aspects. On the other hand, the comprehensive assessment with several self-report questionnaires might have a negative effect on trial adherence, increasing attrition rates. These not yet well examined and understood research biases on intervention effects inherent to RCTs need to be taken into account when interpreting the coming effectiveness findings.

In conclusion, Internet- and mobile-based interventions might have the potential to augment health care services substantially, as they could be a temporal and local flexible, accessible, and cost-effective treatment alternative. AYA with chronic somatic diseases and comorbid anxious and/or depressive symptoms could be supported by iCBT to reduce mental disturbances. With iCBT as a low-threshold and low-intensity intervention, progress and chronification of mental disorders might be preventable at an early stage. The first results of youthCOACH_CD_ are expected to be available in 2021.

### Trial status

Protocol version number 1.0 (submitted on 22/08/2019). Recruitment start is scheduled for October 2019 and will be expected to complete in April 2021.

## Supplementary information

**Additional file 1.** SPIRIT 2013 Checklist: Recommended items to address in a clinical trial protocol and related documents.

## Data Availability

Individual participant data will be made available on request after de-identification beginning 12 months following article publication of the effectiveness and the cost-effectiveness paper. Data will be made available to researchers who provide a methodologically sound proposal, not already covered by others. Proposals should be directed to the corresponding author. Data requestors will need to sign a data access agreement. Provision of data is subject to data security regulations. Investigator support depends on available resources.

## Abbreviations

ATQ-R: Automatic Thoughts Questionnaire-Revised
AYA: Adolescents and young adults
BADS: Behavioral Activation for Depression Scale
BDI-II: Beck Depression Inventory-Revised
BSSS: Berlin Social Support Scales
CAMHSRI-DE: Child and Adolescent Mental Health Services Receipt Inventory
CATS: Child and Adolescent Trauma Screen
CBT: Cognitive behavioural therapy
COACH: *C*hronic C*o*nditions in *A*dolescents: Implementation and Evaluation of Patient-centred *C*ollaborative *H*ealthcare
CODI: Coping with a Disease (questionnaire for children and adolescents with chronic health conditions)
CSQ-I: Client Satisfaction Questionnaire adapted to Internet-based interventions
EQ-5D-Y: EuroQol Five-Dimensional Questionnaire-Youth
GAD-7: Generalized Anxiety Disorder Screener
GSE: General Self-Efficacy Scale
iCBT: Internet- and mobile-based cognitive behavioural therapy
ICC: Intra-class correlation
ICER: Incremental cost-effectiveness ratio
INEP-On: Inventory for recording negative effects of online interventions
ITT: Intention-to-treat
IUES: Internet-Use Expectancies Scale
MFQ: Mood and Feelings Questionnaire (caregiver)
PHQ-9: Patient Health Questionnaire
SCARED: Screen for Child Anxiety Related Emotional Disorders (caregiver)
SD: Standard Deviation
SRGS: Stress-Related Growth Scale
TAU+: Treatment as usual+
WAI-SR: Working Alliance Inventory-Short Revised
youthCOACH_CD_: youthCOACH_chronic disease_
